# Do-It-Yourself Ultrasonography Phantom Model for Improving Hand Skills: A Technical Report

**DOI:** 10.7759/cureus.54750

**Published:** 2024-02-23

**Authors:** Rishi Anand, Bhanu Pratap Swain, Deb Sanjay Nag, Roushan Patel, Roshan Lal Gope

**Affiliations:** 1 Anaesthesiology, Tata Main Hospital, Jamshedpur, IND; 2 Anaesthesiology, Manipal Tata Medical College, Jamshedpur, IND

**Keywords:** simulation design, simulation in medical education, ultrasound training, ultrasound probe training, phantom model, water phantom, ultrasonography

## Abstract

This technical report focuses on developing a do-it-yourself (DIY) model of a water phantom for training in ultrasound-guided needle insertion techniques. Ultrasound technology is becoming more widely used in perioperative and intensive care settings. However, accurate needle placement using ultrasound guidance necessitates strong spatial reasoning and hand-eye coordination. To address this, the authors experimented with a water phantom model that is cost-effective, easily accessible, and efficient for training. The DIY water phantom was made using materials such as an examination glove, a used vial rubber cap, water, adhesive tape, sealing glue, and a target object. This technical report discusses the process of assembling the water phantom and the potential benefits it offers for ultrasound training.

## Introduction

Ultrasonography technology has greatly improved the accuracy and reduced complications associated with performing blocks and invasive procedures. The increasing availability, decreased costs, and improved portability of USG equipment have led to its growing use in perioperative and intensive care settings. However, achieving precise ultrasound-guided needle placement requires strong spatial reasoning and hand-eye coordination [1]. A water phantom is a device used in ultrasound training to simulate the acoustic properties of human tissue. It is a container filled with water and usually includes various inserts that mimic different types of tissue, such as muscle, fat, and bone [2]. The water phantom is used to simulate a human body and to provide an environment for hands-on training in ultrasound techniques, such as image acquisition and interpretation. The use of phantoms in ultrasound training allows learners to practice scanning techniques in a safe, controlled environment without the risk of harm to patients [2]. To enhance proficiency and ensure patient safety before actual interventions, training on ultrasound phantoms is recommended [2]. The ideal USG phantom should replicate human tissue in echogenicity, allow for multiple uses, be cost-effective and simple to create, provide tactile feedback, and pose no health risks. Various types of phantoms, including water, gelatin agar, blue, meat, and cadavers, have been experimented with and described in the literature [2]. The authors concentrated on the water phantom model, as this is cost-effective, easy to prepare, lacks biohazards, and offers good echogenicity. Water leakage during needling is a common problem associated with USG water phantom. The proposed water phantom overcomes this issue with the introduction of a sealing mechanism using a vial rubber stopper cap fixed to a rubber glove.

## Technical report

The authors experimented with the water phantom model as it is an easy-to-prepare, do-it-yourself (DIY) project that is cost-effective, readily available, time-saving, and capable of displaying good echogenicity. However, water models have been associated with issues such as water leakage, limited tactile feedback, poor needle holding, and poor resemblance to human tissue in echogenicity [2]. With the introduction of a sealing mechanism with a rubber stopper cap attached to a rubber glove, the authors have attempted to improve needle holding, provide better tactile feedback during insertion, and decrease water leakage, allowing longer needling practice.

**Figure 1:**
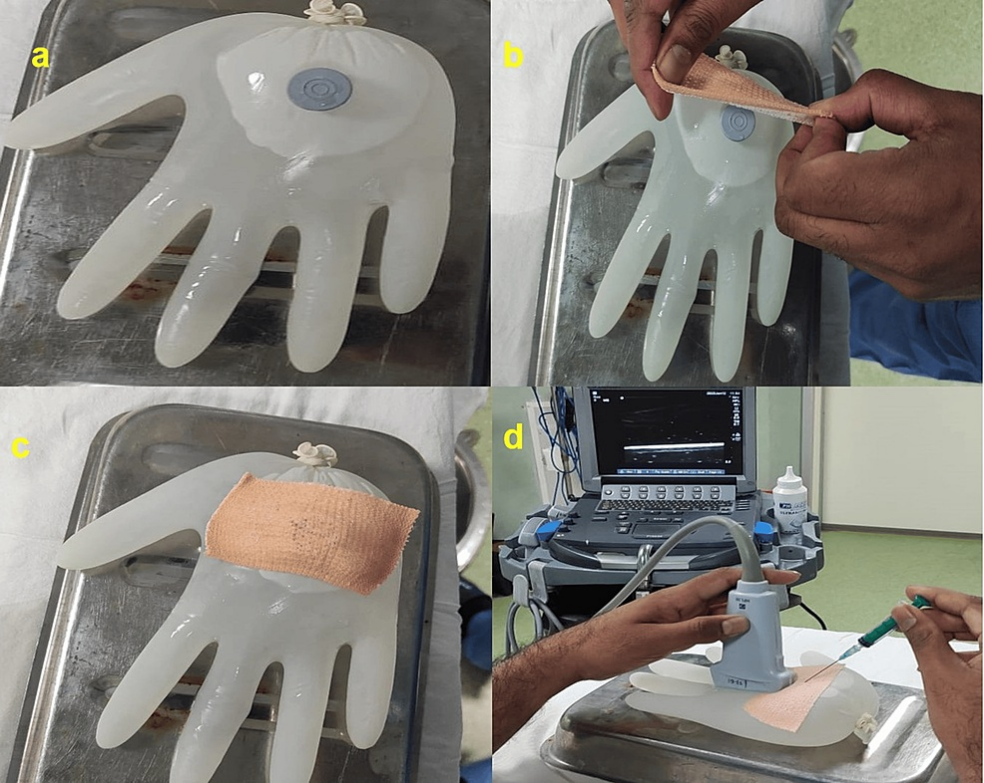
Assembly of the water phantom model (a) The used vial rubber stopper is glued to a water-filled rubber glove; (b) and (c) The rubber stopper site has been further reinforced with adhesive tape; (d) The ultrasound probe is placed close to this site and a needle is introduced through the rubber stopper. The operator is supposed to visualise the needle using ultrasound to improve hand-eye coordination.

**Figure 2:**
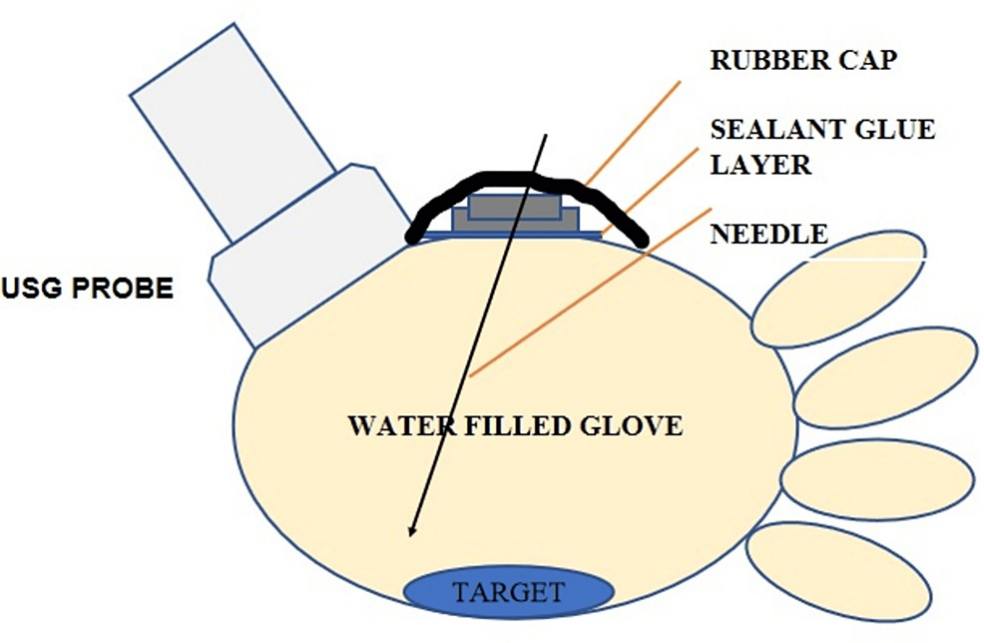
A schematic diagram of the water phantom model The image has been created by the authors.

The authors created a DIY USG water phantom with easily available materials, including an examination glove, a used vial rubber cap, water, adhesive tape, sealing glue, and a target object (e.g., a coin). The assembly process, as shown in Figure 1, entails filling the examination gloves with water, attaching the rubber cap of a used vial to the surface, using rubber-based adhesive glue and tape, and establishing a puncture site. A rubber cap attached to the glove surface improves sealing around the puncture site, helps in holding the needle during manoeuvres, and improves tactile feel during training. This allows the use of the phantom prototype for a longer duration. Additionally, a target material, such as a metal coin, can be positioned. A schematic diagram is shown in Figure 2.

**Figure 3:**
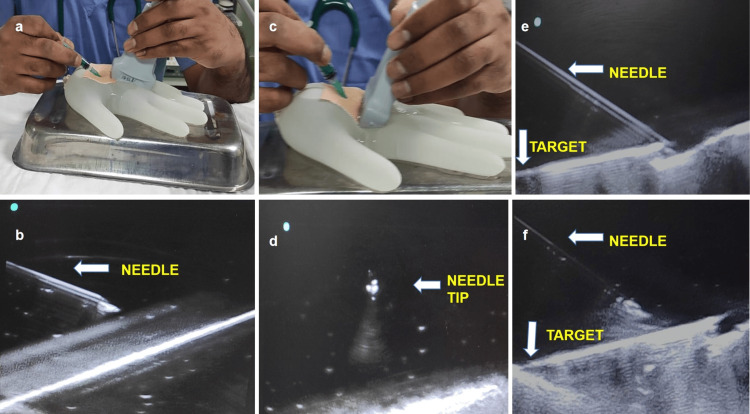
Performance of manoeuvres under ultrasound vision The manoeuvres to be practised are as follows: (a) and (b) In-plane visualisation and advancement of the needle up to the target under ultrasound vision; (c) and (d) Out-of-plane visualisation of the needle tip hitting a target object under ultrasound; (e) and (f) Moving the needle from one end to the other end of a target object under ultrasound vision.

The USG probe with gel should be positioned near the specified puncture site, and the manoeuvres to be practised involve visualising and advancing the needle in-plane up to the target under vision, visualising the needle tip out-of-plane to hit the target object, and moving the needle from one end to the other end of the target object under vision. These manoeuvres have been shown in Figure 3. The authors were able to perform needling more than five times and the phantoms maintained their shape for more than 30 minutes. This phantom is for single-session use only.

## Discussion

Anaesthesiologists are using ultrasound more often for both interventional and diagnostic procedures in critical care and perioperative care. These days, many anaesthetic procedures, including vascular access and regional anaesthetic blocks, are carried out under USG supervision [3]. Efficient and procedural safety is enhanced by real-time visualisation of the needle. Adept hand-eye coordination and psychomotor skills are necessary for a safe approach and needle visualisation; trainees must practice this technique. Ultrasound-guided procedures are taught to trainees in several centres by direct observation of patient procedures [4].

To enhance training and lessen complexity, the focus has shifted in recent years to simulation-based training. In the past, a lot of ultrasound phantoms were created to enhance hand skills [2]. It has been demonstrated that phantom-based simulations enhance technical procedural skills, lower anxiety, boost confidence, lower the risk of potential complications, and improve the proficiency of novices performing ultrasound-guided procedures. Additionally, they offer a safe setting for trainees to become acquainted with focused ultrasound-guided treatments [4,5].

However, the expense of high-fidelity simulators and phantoms may be unaffordable for institutions with little funding, particularly in developing countries with low per capita income [6]. A number of researchers have shared their simple, affordable, and high-fidelity ultrasound simulation phantom models [6-8].

After conducting a literature review and assessing local institutional needs, the authors decided to design a water phantom prototype from easily available waste materials inside the operation theatre. In order to prepare the phantom prototype, we chose to concentrate on local availability, simple handling, and quick preparation. The authors created and tested this model on routine needle manoeuvres in order to assess its utility. Given its benefits, such as its simplicity of preparation, accessibility to required materials, affordability, excellent needle visualisation, lack of biohazard, and adaptability in terms of target and model shape, we are satisfied with the do-it-yourself USG water phantom model. Innovative modifications like the use of a used vial rubber stopper not only help in limiting water leakage during needling but also provide tactile feedback during needle advancement.

Our phantom model's drawbacks include its short lifespan, poor human tissue replication, one-dimensional tactile sensitivity limitation, low depth suitability, poor clinical scenario functionality, and limited supply of water-resistant rubber-based sealing glue.

The authors also suggest potential enhancements to deal with its shortcomings. To better simulate human tissue, users can add psyllium to water. Other hollow rubber items can be used in place of gloves to allow practice with needle depth. Moreover, additional innovation on the target object could be used to mimic the placement of needles in anatomical structures, nerve bundles, or vessels. The authors believe this prototype will be able to meet the training needs of beginners and, because of its adaptability, will enable future refinement based on input from large-group experiences.

## Conclusions

In summary, the development of a low-cost USG phantom through innovation offers a promising way to improve training and competency in USG-guided procedures in settings with limited resources. It might aid inexperienced individuals' training prior to patient exposure, improving patient outcomes and safety. Water phantoms have historically been considered inadequate for USG-guided needle placement procedure simulation. The proposed water phantom model overcomes a few of these deficiencies and should facilitate needle advancement and handling practices to enhance dexterity and hand-eye coordination. However, further studies and large-scale trials are necessary to establish its utility and efficiency with respect to other phantom models in USG training.

## References

[1] Mendiratta-Lala M, Williams T, de Quadros N, Bonnett J, Mendiratta V (2010). The use of a simulation center to improve resident proficiency in performing ultrasound-guided procedures. Acad Radiol.

[2] Kim YH (2016). Ultrasound phantoms to protect patients from novices. Korean J Pain.

[3] Gupta PK, Gupta K, Dwivedi AN, Jain M (2011). Potential role of ultrasound in anesthesia and intensive care. Anesth Essays Res.

[4] Giannotti E, Jethwa K, Closs S, Sun R, Bhatti H, James J, Clarke C (2022). Promoting simulation-based training in radiology: a homemade phantom for the practice of ultrasound-guided procedures. Br J Radiol.

[5] Torrano V, Zadek F, Bugada D (2022). Simulation-based medical education and training enhance anesthesia residents’ proficiency in erector spinae plane block. Front Med (Lausanne).

[6] Palmer JM, Little A, Tran QV (2022). Cost-effective training models in point-of-care ultrasound for medical students in emergency medicine: an evaluation of current resources. Cureus.

[7] Bude RO, Adler RS (1995). An easily made, low-cost, tissue-like ultrasound phantom material. J Clin Ultrasound.

[8] Kocharyan H, Kallini J, Aida SK, Harvill M (2018). A cost-effective alternative formulation of ultrasound phantom for vascular access instruction: cost-effective hands-on procedural training. J Vasc Access.

